# Measuring the Quality of Observational Study Data in an International HIV Research Network

**DOI:** 10.1371/journal.pone.0033908

**Published:** 2012-04-06

**Authors:** Stephany N. Duda, Bryan E. Shepherd, Cynthia S. Gadd, Daniel R. Masys, Catherine C. McGowan

**Affiliations:** 1 Department of Biomedical Informatics, Vanderbilt University School of Medicine, Nashville, Tennessee, United States of America; 2 Department of Biostatistics, Vanderbilt University School of Medicine, Nashville, Tennessee, United States of America; 3 Division of Infectious Diseases, Department of Medicine, Vanderbilt University School of Medicine, Nashville, Tennessee, United States of America; Statens Serum Institute, Denmark

## Abstract

Observational studies of health conditions and outcomes often combine clinical care data from many sites without explicitly assessing the accuracy and completeness of these data. In order to improve the quality of data in an international multi-site observational cohort of HIV-infected patients, the authors conducted on-site, Good Clinical Practice-based audits of the clinical care datasets submitted by participating HIV clinics. Discrepancies between data submitted for research and data in the clinical records were categorized using the audit codes published by the European Organization for the Research and Treatment of Cancer. Five of seven sites had error rates >10% in key study variables, notably laboratory data, weight measurements, and antiretroviral medications. All sites had significant discrepancies in medication start and stop dates. Clinical care data, particularly antiretroviral regimens and associated dates, are prone to substantial error. Verifying data against source documents through audits will improve the quality of databases and research and can be a technique for retraining staff responsible for clinical data collection. The authors recommend that all participants in observational cohorts use data audits to assess and improve the quality of data and to guide future data collection and abstraction efforts at the point of care.

## Introduction

Accurate and valid HIV research results depend on high-quality clinical and laboratory data. Excellent patient care itself depends on accurate recording and transcription of such information [1]. Interventional clinical trials, such as those generating data for pre-marketing approval by regulatory agencies or those conducted by the AIDS Clinical Trials Group [2], follow careful data quality assurance procedures. This ensures that investigators comply with the International Conference on Harmonization's guidelines for Good Clinical Practice and that collected data reflect true measurements [3], [4]. A data audit, in which an external review team compares a research dataset to the original data collection documents, is the standard method of assessing the quality of data in clinical trials [5]–[8].

Depending on the nature of the research question, researchers and funding agencies may use routine patient care data as a readily available and inexpensive supplement or alternative to data generated through prospective clinical trials [9], [10]. Databases that pool such observational data from multiple, international sites have become particularly important resources for HIV/AIDS research due to increased interest in measuring global trends in the epidemic and the side effects and long-term outcomes of antiretroviral (ARV) therapy [11]–[15]. Routine medical care data, however, do not undergo the same stringent quality controls commonly applied in clinical trials. International multi-center HIV networks that use routine patient care data may be at higher risk of having data quality issues because monitoring for quality at geographically distant locations is difficult, time-consuming, and expensive.

Many networks that repurpose routine medical care data for research rely on data cleaning and cross-referencing performed at the data coordinating center [16]. These techniques can confirm the data's internal consistency and identify missing values but cannot determine data accuracy and authenticity. Comparing research data to their source documents through audits is therefore an essential additional step in verifying the data's accuracy [17]. Despite the importance of using high-quality data, no multi-center observational HIV cohort has published research indicating that they conducted frequent source-to-database data audits. As a result, the quality of data and the accuracy of results from many multi-site HIV cohorts can be uncertain.

We evaluated the accuracy and completeness, as assessed by on-site data audits, of routine patient care data submitted for research by seven sites participating in an international collaborative HIV research network: the Caribbean, Central and South America Network for HIV Epidemiology (CCASAnet).

## Methods

### Network description

CCASAnet is one of seven regional networks that belong to International Epidemiologic Databases to Evaluate AIDS (IeDEA), an international, multi-center research consortium funded by the U.S. National Institutes of Health [13]. The CCASAnet project aims to create a shared repository of HIV data from participating clinics and hospitals in Latin America and the Caribbean, to use the combined data to examine regional HIV epidemic characteristics, and to contribute to worldwide IeDEA studies [18], [19]. Seven urban HIV/AIDS treatment centers participate in the network, including sites in Argentina, Brazil, Chile, Haiti, Honduras, Mexico, and Peru, and the network is overseen by a data coordinating center (DCC) at Vanderbilt University in Nashville, Tennessee, USA. To preserve the anonymity of the clinics, we have labeled them randomly as sites A–G.

All seven CCASAnet sites used paper medical records as the primary means of storing patient information. The paper clinical records generally contained a structured patient intake form and handwritten follow-up visit notes. Nursing staff at sites D, F, and G maintained detailed drug dispensing forms that were kept with the patient chart and used to verify drug prescriptions and dates. The different data collection practices and resources at each audit site are detailed in Table 1.

**Table 1:** Characteristics of data collection, abstraction, and management at audit sites A–G.

	Sites						
Characteristic	A	B	C	D	E	F	G
Structured patient follow-up visit form	Yes	no	no	no	no	no	no
Drug dispensing form	no	no	no	Yes	no	Yes	Yes
Electronic laboratory system	no	Yes	Yes	no	no	Yes	no
On-site research database (vs. commercially hosted and managed database)	Yes	Yes	Yes	Yes	no	Yes	Yes
Data manager	Yes	Yes	Yes	Yes	no	no	Yes
Full-time data abstraction team	no	no	no	Yes	no	no	Yes
Internal data audits	no	no	no	no	no	Yes	no

At all sites, a team of clinicians, data entry personnel, or administrative staff abstracted information from the paper medical record and entered the results into an electronic research database from which data were extracted for CCASAnet studies. Sites B, C, and F had access to electronic laboratory systems that exported test results directly into their research databases. The majority of these databases were designed and maintained by local staff and implemented in Microsoft Access. One site used a commercial data warehousing service in place of an on-site database server. Two of the seven sites employed experienced data managers (B, C); three of the remaining sites had data managers without formal training (A, D, G.) Only Site C operated an extensive data center. Site F was the only site that actively conducted internal quality reviews of their research data.

The Vanderbilt University Medical Center Institutional Review Board approved this project. Local centers de-identified all data before transmitting it to the DCC, so no informed consent was required.

### Data audit preparation and process

Between April 2007 and March 2008, a team from the DCC – including at least one HIV clinician and one informaticist – conducted on-location audits of the datasets received from CCASAnet member sites. Our audit techniques involved verifying data integrity using source documents and were adapted from those used in clinical trials to ensure Good Clinical Practice (GCP) compliance [3].

The DCC data manager selected approximately 30 records from each site's database using simple random sampling, so that within a site's dataset, each record had an equal probability of being selected. We sent research identification numbers (IDs) for the first 20 randomly selected records to the site ten days before the data monitoring visit to allow local data personnel to retrieve the records in advance. We requested the remaining ten records from the site investigators on the first audit day.

Our initial audits lasted two to three days per site. Sites for which we recommended major quality interventions were re-audited during the current or subsequent audit cycle. During each audit, we compared the contents of the study database to the local source documents and noted discrepancies and inconsistencies in individual data elements. The available source documents included paper clinical records and, where available, electronic laboratory, pharmacy, and medical record reports. We reviewed as many source documents as the sites could locate during the visit and consulted local site personnel for clarifications as needed. Audit findings were recorded on a structured paper audit form and later entered into an Excel spreadsheet for analysis.

The audited variables included those most relevant to proposed consortium studies: patient demographics, HIV-related risk factors, weight measurements, CD4^+^ lymphocyte (CD4) counts, plasma HIV-1 RNA levels (viral load), all ARV regimens, and all dates associated with each measurement. When a patient's ARV regimen was recorded as “current” or “ongoing” in the database, we verified that the patient was still taking the specified drug combination around the time the site data were submitted to the DCC.

### Error classification

After each audit, we reviewed the completed paper audit forms and categorized audit results using standardized audit codes from the European Organization for the Research and Treatment of Cancer (EORTC) [6]. The audit data were labeled correct (code 1) if the database values submitted to the DCC matched the values in the paper clinical record or other on-site source documents. Major errors (code 3) represented discrepancies between the clinical record and database values that the physician on the audit team deemed clinically meaningful. Other discrepancies were labeled minor errors (code 2, e.g., weight values rounded to the nearest integer, dates in the database within six days of the documented date). Missing/missed data (code 4) included values for requested information (e.g., baseline weight) that had been left blank in the database. Values that existed in the submitted database but not in the clinical record were labeled sourceless (code 5). One auditor performed the initial classification, a second auditor reviewed all classifications, and all disagreements were resolved by joint record review. A coded sample record is presented in Table S1.

### Statistical analysis

We calculated error rates by dividing the number of erroneous clinical record/database value pairs (codes 3, 4, and 5) by the number of audited value pairs. We compared error rates across variables using a generalized linear mixed model to account for correlations between variables from the same record and within the same clinic. Analyses comparing major error rates (code 3) between variables did not include missing or sourceless errors (codes 4 and 5) in the denominator.

## Results

We requested a total of 208 randomly selected patient records during seven data audits at Sites A–G. Of these 208 records, 16 could not be located or were unavailable because they were needed for patient care. The majority of missing records (11 of 16) were from Site A. We reviewed 184 of the remaining 192 charts (eight charts were not audited because of time constraints) comprising 4,223 unique data points. The number of unavailable, available, and audited charts by site is shown in Table 2.

**Table 2:** Availability of randomly selected clinical records requested by the audit team according to site.

Audit Site	Total charts requested	Charts available and audited	Charts available but not audited	Charts unavailable
A	40	29	0	11
B	28	23	3	2
C	17	17	0	0
D	28	27	0	1
E	33	27	5	1
F	35	35	0	0
G	27	26	0	1
**Total**	**208**	**184**	**8**	**16**

The dataset of all audit results contained 3,581 correct data points, 66 minor errors, 171 major errors, 274 missing values, and 131 sourceless values. Minor errors – which were not counted towards error rates – included dates that were shifted by a few days (45%), inappropriately rounded weight measurements (36%), and weight, CD4, and viral load values where a probable typographical slip resulted in small and clinically insignificant value differences (19%). All audited instances of patient gender were correct, and all birth dates were correct at four of seven sites. At the remaining three sites, 7–19% of the birth dates recorded in the site database differed by a week or more from values in the clinical record. Weight values and their associated dates were entered in the database with few or no errors at Sites F and G. Error rates at the remaining sites ranged from 11–93% for weight measurements and 15–100% for weight dates. At the three sites with the highest error rates, weight data missing from the database were the primary cause of error (24–89% missing weight measurements and 24–100% missing weight dates).

Sites varied less in error rates for laboratory values: CD4 count (1–21%), CD4 dates (1–27%), viral load (1–42%), and viral load dates (0–42%). For the records with major errors, the median and interquartile range (IQR) for absolute differences between the database and chart values were 20 cells/mm^3^ (9–101 cells/mm^3^) for CD4 count, 23 days (10–56 days) for CD4 date, 65,200 copies/ml (47,000–145,600 copies/ml) for viral load, and 22 days (11.5–50 days) for viral load dates. Table 3 shows the number of variables audited at each site and their specific error rates.

**Table 3:** Total number of audited variables and percentage of erroneous data by data type during initial audits at seven sites^a^.

	Audit Sites^b^															
	A		B		C		D		E		F		G		All	
	*N*	%err	*N*	%err	*N*	%err	*N*	%err	*N*	%err	*N*	%err	*N*	%err	*N*	%err
**Variables**																
Gender	29	0%	23	0%	17	0%	27	0%	27	0%	35	0%	26	0%	184	0%
Birth date	29	7%	23	9%	17	0%	27	19%	27	0%	35	0%	26	0%	184	5%
Weight	29	31%	37	41%	55	11%	26	38%	27	93%	268	1%	45	2%	487	14%
Weight date	29	21%	37	30%	55	15%	26	38%	27	100%	268	0%	45	9%	487	14%
**Laboratory data**																
CD4	29	14%	33	21%	31	6%	96	13%	132	5%	134	1%	88	5%	543	7%
CD4 date	29	21%	33	27%	31	10%	96	16%	132	17%	134	1%	88	8%	543	12%
Viral load^c^	29	7%	26	42%	0	–	57	25%	120	7%	112	1%	84	4%	428	9%
Viral load date^c^	29	17%	26	42%	0	–	57	28%	119	13%	112	0%	84	7%	427	12%
**Antiretroviral regimen data**																
Regimen	46	11%	54	26%	23	13%	38	21%	49	22%	67	7%	47	19%	324	17%
Start date	46	28%	54	56%	23	13%	38	32%	49	39%	67	12%	47	26%	324	30%
Stop date	30	27%	54	50%	7	29%	38	29%	49	33%	67	10%	47	38%	292	30%
**All**	354	17%	400	34%	259	10%	526	21%	758	20%	1299	2%	627	10%	4223	14%

a This table shows the number of variables audited in each of eleven categories of data, including gender, birth date, weight, CD4 count, viral load, antiretroviral (ARV) regimens, and all associated dates.

b Columns contain the counts for each site (*N*), along with the percentage of data that was labeled “in error” by auditors (%err). The reported percentage of erroneous data includes incorrect, missing, and sourceless values (error categories 3, 4, and 5), but not minor errors.

c Site C did not submit any viral load data.

**Figure 1:**
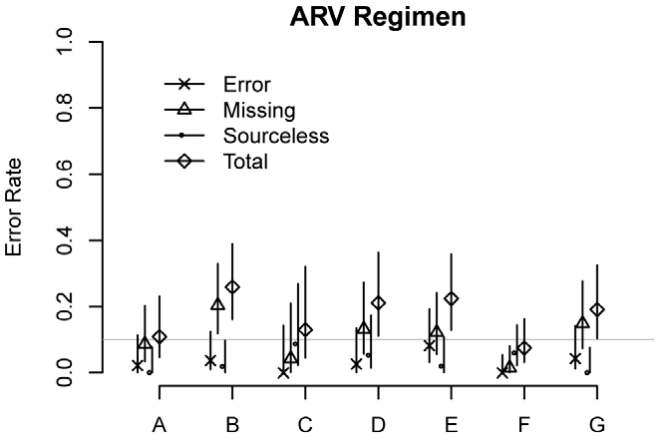
Error rates by error type for antiretroviral regimens. The chart shows the rates of overall, incorrect, missing, and sourceless data errors and their 95% confidence intervals for antiretroviral (ARV) regimens. The horizontal line represents error rates of 10%.

**Figure 2:**
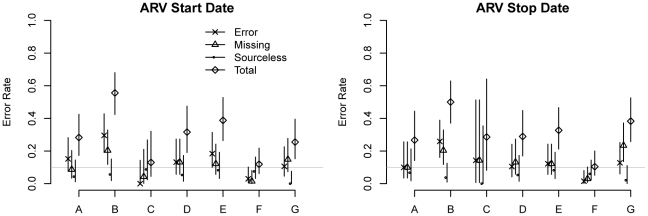
Error rates by error type for start and stop dates of antiretroviral treatment. The two tiled charts show the rates of overall, incorrect, missing, and sourceless data errors and their 95% confidence intervals for the start and stop dates of patients' antiretroviral regimens. The horizontal line represents error rates of 10%.

Errors in antiretroviral data according to audit site and error type (and their 95% confidence intervals) are shown in Figure 1 (regimen data) and Figure 2 (dates). For 7–26% of ARV regimens, the drug combinations were missing from the site's submitted database, incorrectly entered into the database, or not substantiated by content in the clinical record. Error rates for regimen start and stop dates ranged from 10–56%. There was no difference in error rates when comparing stopping and starting dates (*P*>0.25 for both major errors alone and overall errors.) For records with major errors, the median absolute difference between start/stop dates found in the chart and those recorded in the database was 88 days (IQR: 31–365 days).

Overall, the error rates for ARV regimen start and stop dates were higher than the error rates for all other non-ARV dates, including the dates of weight, CD4 count, and viral load measurements (*P*<0.001 for all.) For CD4 count, viral load, and ARV data, the associated dates had a higher rate of major errors (category 3) than the actual values (*P*<0.001 for all sites), whereas weight values had more major errors than weight dates (*P* = 0.028 for all sites).

After the findings of the initial audit, Site B cleaned and reabstracted their study data and quickly submitted an updated version, allowing us to reaudit the site at the end of the initial audit cycle. We reviewed 26 randomly selected records with 463 variables during this second audit at Site B; four additional records we requested were not available during the audit period. We observed reduced error rates in all variable categories. The overall error rate dropped from 34% to 17% (Table 4).

**Table 4:** Variable counts and error rates by data category during initial and follow-up audits at a single site.

	InitialSite Audit		Follow-up Site Audit	
	*N*	%err	*N*	%err
**Variables**				
Gender	23	0%	26	0%
Birth date	23	9%	26	8%
Weight	37	41%	42	26%
Weight date	37	30%	42	21%
**Laboratory data**				
CD4	33	21%	35	6%
CD4 date	33	27%	35	6%
Viral load	26	42%	32	16%
Viral load date	26	42%	32	13%
**Antiretroviral regimen data**				
Regimen	54	26%	65	12%
Start date	54	56%	64	23%
Stop date	54	50%	64	33%
**All**	400	34%	463	17%

## Discussion

Our data audits revealed substantial error rates in data submitted by all seven participating clinics. The majority of errors were due to measurements found in clinical records that were not entered into the database, laboratory values with no source documents, and incorrect antiretroviral regimens. Dates were especially prone to error, and sites had the most difficulty accurately capturing antiretroviral drug regimens and their associated dates. Most sites had error rates above 10% for ARV regimens and dates. These findings would trigger strict quality interventions in prospective clinical trials, which typically require fewer than 50 errors per 10,000 fields (<0.5% error rate) [20]. In the context of clinical trials, however, source-to-database audits like those described here generally report similarly high error rates when compared to case-report-form-to-database audits [21].

We found that data inconsistencies resulted from how the sites recorded information in the clinical record, how they abstracted data for research, and how they entered, stored, and formatted the data in the electronic database. Many laboratory dates could not be confirmed because the original laboratory reports had been discarded, a common practice due to lack of storage space. Errors in ARV data often resulted from haphazard data abstraction from paper records used for clinical care. Sites that used rotating personnel for data abstraction, such as care providers, medical students, residents, and other trainees, appeared to have higher rates of ARV data errors compared with those that assembled focused and well-trained teams. Error rates did not appear to be associated with the level of experience of the local data management team or with the presence of a data center. We currently are performing additional studies to better understand reasons for data inconsistencies.

The audit functioned as a useful data quality control for both the data coordinating center and the participating sites. It allowed the DCC to identify and resolve weaknesses in submitted data before erroneous data could affect study results and provided sites with a baseline estimate of their data quality. As a result, all published CCASAnet studies use revised site data.

The findings prompted us to recommend many of the same quality improvement interventions for each site:

- **Standardize data abstraction, database entry procedures, and personnel training to reduce variability in data quality.** The audit team observed avoidable errors like improperly selected laboratory dates (laboratory results should be paired with the date the sample was drawn rather than the date it was processed or the date of the finalized laboratory report) and rounded weight values (i.e. 56.7 kg rounded to 56 kg) that led to overall information loss. Such systematic errors could be prevented by educating data abstractors to follow consistent rules during data collection and entry.
- **Develop structured patient visit forms, either paper or electronic, to encourage consistent provider documentation and reduce the amount of missing information.** Our audits determined that the majority of weight-related errors from three sites were due to missing weight measurements. By replacing the blank sheets of paper these sites used for clinician notes with a printed form that prompted providers to record patient weight in a field, some of these missing values could be prevented.
- **Retain laboratory reports whenever possible to reduce the number of sourceless, unverifiable laboratory values and dates.** Sites D, E, and G did not routinely keep copies of the original laboratory reports in the patient medical record, resulting in code 5 errors for associated values (sourceless data). The audit team compared the study data to handwritten laboratory flowsheets when possible, but these were not true source documents.
- **Revise the procedure for storing dates in the database so that data abstractors can accurately record dates that are only known to month or year precision.** We noted that ambiguous dates in the patient record resulted in frequent date-related errors in ARV information.

Many of these recommendations are based on standard data management and clinical trials practices [22]. Each site positively accepted the feedback and submitted a data quality improvement plan to the DCC. We have not yet formally studied the impact of these quality improvement interventions, but early results suggest that the audit process has led to improved site procedures. Our first follow-up audit found a 50% decrease in the overall error rate, with most of the remaining errors resulting from missed data (values that existed in the clinical chart but were not entered in the database) rather than incorrect information.

Our study had both strengths and weaknesses. The analysis of audit findings used a straightforward error categorization system that required little subjective interpretation, making these findings easy to replicate. Our multi-lingual auditors were confident reviewing medical records in French, Spanish, and Portuguese, and their training allowed them to identify causes of error related to clinical process and data handling. Potential variation was minimized by using the same core audit team during every visit. However, the audit team could inspect only a fraction of records at each site, so although records were randomly selected for auditing, the true error rates may differ from the estimated rates reported here. Furthermore, the audit process evolved as the team gained experience with each successive audit, so audits performed later may have been more likely to uncover errors.

Audit results might have been more accurate if auditors had requested not just a third, but all records on the date of the audit visit, to discourage sites from reviewing and potentially editing records in advance. Such an approach, however, might increase the frequency of selected records being unavailable for audit. We did not notice any difference in results between records requested on the date of the audit visit compared to those requested ten days in advance.

Routine clinical care records are a valuable source of diverse, plentiful, and relatively inexpensive medical data for HIV/AIDS research. Without quality control, however, these data may not be sufficiently complete or reliable for research. Investigators who reuse clinical care data must be proactive in addressing potential quality concerns. To our knowledge, we are the first multi-site observational HIV cohort that has performed source-to-database data quality audits. We do not suspect that the error rates observed in our cohort are substantially higher than those that would be seen if source-to-database audits were performed in other multi-center HIV cohorts. Indeed, several of our sites have participated in other multi-site cohorts such as ART-LINC, TCHARI, and CHIAC [14], [23], [24]. We do not claim that the findings of other multi-center observational HIV cohorts are erroneous, but it is difficult to interpret study validity without a formal assessment of data quality. Collaborative research networks – especially those in international settings – should strongly consider implementing formal audit programs to evaluate the reliability of their data sources, to correct discrepancies in data that have already been collected, and to prevent errors in prospective data collection.

On-location audits require often-scarce resources. In order to minimize costs, many of our audits were performed during visits with other scientific objectives. We are currently developing an electronic audit support tool to help standardize and simplify the audit process, allowing auditors to import an electronic dataset, select a set of records to audit, document their findings in real-time using a pre-defined error taxonomy, and quickly generate summaries of audit results. We also are exploring the possibility of incorporating data quality self-assessments as a formal component of our data quality control procedures. With such an approach, sites can perform self-assessments of data quality, which may permit the DCC to reduce the frequency of external audits. Under certain conditions, audit results can be used to statistically adjust estimates based on the original, error-containing data [25]. A data audit should be viewed as an important tool for improving the quality of data, the validity of associated study results, and the reliability of future data collection procedures.

## Supporting Information

Table S1 **Example of audit error coding in a comparison of a patient's antiretroviral drug regimens as recorded in the database with those found in clinical records.**
(DOCX)
