# Supplementary material for: Measuring the Quality of Observational Study Data in an International HIV Research Network
Source: PLoS One. 2012 Apr 6;7(4):e33908. doi: 10.1371/journal.pone.0033908 (PMC3320898; doi:10.1371/journal.pone.0033908)
Supplement: Table S1 — Example of audit error coding in a comparison of a patient's antiretroviral drug regimens as recorded in the database with those found in clinical records. (DOCX) [file pone.0033908.s001.docx]

Table S1: Example of audit error coding in a comparison of a patient’s antiretroviral drug regimens as recorded in the database with those found in clinical records

| **Test Record #1** | **Value in database** | **Value in clinical record** | **Comments** | **Audit Code** |
| --- | --- | --- | --- | --- |
| **Demographics** | | | | |
| Gender | Male | Male |  | correct (1) |
| Birthdate | 1973-01-31 | 1973-01-31 |  | correct (1) |
| Weight | 56 | 56.5 | Rounding | minor error (2) |
| Weight date | 24 Aug 2002 | 24 Aug 2002 |  | correct (1) |
| **Laboratory data** | | | | |
| CD4 | --- | 110 |  | missing (4) |
| CD4 date | --- | 15 Aug 2002 |  | missing (4) |
| Viral load | 32,000 | 320,000 | Probable typo | incorrect (3) |
| Viral load date | 15 Aug 2002 | 15 Aug 2002 |  | correct (1) |
| **Antiretroviral regimen data** | | | | |
| Regimen 1 | AZT DDI RIT SAQ | AZT DDI RIT SAQ |  | correct (1) |
| Start date | 28 Aug 2002 | 28 Aug 2002 |  | correct (1) |
| Stop date | 09 May 2006 | 06 Feb 2005 | Notes from the HIV clinic show that regimen 1 was stopped on 06 Feb 2005. Only the Tuberculosis clinic continues to report regimen 1 active until 09 May 2006. | incorrect (3) |
| Regimen 2 | --- | AZT 3TC EFV |  | missing (4) |
| Start date | --- | 06 Feb 2005 | (documented in hospital orders and notes from HIV clinic) | missing (4) |
| Stop date | --- | 01 Jan 2006 |  | missing (4) |
| Regimen 3 | --- | D4T 3TC EFV KAL |  | missing (4) |
| Start date | --- | 14 Feb 2006 | Regimen 3 began during hospitalization and is listed as dispensed on hospital orders checksheet | missing (4) |
| Stop date | --- | 21 Feb 2006 | Regimen 3 stops appearing in hospital orders. | missing (4) |
| Regimen 4 | D4T 3TC KAL | D4T 3TC KAL |  | correct (1) |
| Start date | 09 May 2006 | 22 Feb 2006 | Regimen 4 was begun on 22 Feb 2006 during hospitalization and (according to the order sheets) the drugs were administered. | incorrect (3) |
| Stop date | 19 Sept 2006 | 23 Jul 2006 | Clinic notes show Regimen 4 was stopped on 23 Jul 2006. | incorrect (3) |
| Regimen 5 | AZT 3TC KAL | AZT 3TC KAL |  | correct (1) |
| Start date | 19 Sep 2006 | 18 Sep 2006 | Unclear handwriting. | minor error (2) |
| Stop date | current/ongoing | 11 Dec 2007 | Patient died. | incorrect (3) |
